# Temporal analysis of GBIF data reveals the restructuring of communities following climate change

**DOI:** 10.1111/1365-2656.13854

**Published:** 2022-12-07

**Authors:** Armelle Lajeunesse, Yoan Fourcade

**Affiliations:** ^1^ Univ Paris Est Creteil, Sorbonne Université, Université Paris‐Cité, CNRS, IRD, INRAE, Institut d'écologie et des sciences de l'environnementm IEES Créteil France

**Keywords:** animal, citizen science, climate change, climatic debt, community ecology, GBIF

## Abstract

1. Multiple studies revealed an effect of climate change on biodiversity by investigating long‐term changes in species distributions and community composition. However, many taxa do not benefit from systematic long‐term monitoring programmes, leaving gaps in our current knowledge of climate‐induced community turnover.
2. We used data extracted from the Global Biodiversity Information Facility to characterize community reorganization under climate change for nine animal taxonomic groups (ants, bats, bees, birds, butterflies, earthworms, frogs, rodents and salamanders), which, for most of them, had never been studied before in this regard. Using a presence‐only community temperature index (CTI), reflecting the relative proportion of warm‐ and cold‐adapted species, we tested whether and how species' assemblages were affected by climate change over the last 30 years.
3. Across Europe and North America, we observed an average increase in CTI, consistent with a gradual species turnover driven by climate change.
4. At the local scale, we could observe that the composition of most species assemblages changed according to temperature variations. However, this change in composition always occurred with a lag compared to climate change, suggesting that communities are experiencing a climatic debt. Results suggest that anthropization may play a role in the decoupling between the change in CTI and the change in local temperature.
5. The results of our study highlight an overall thermophilization of assemblages as a response of temperature warming. We demonstrated that this response may exist for a large range of understudied terrestrial animals, and we introduced a framework that can be used in a broader context, opening new opportunities for global change research.

## Graphical abstract

Investigating the impact of climate change is an important topic in ecological research, but it requires long‐term data that may not be available. This study demonstrates that the restructuring of species' assemblages in response to climate change can be revealed from unstructured occurrence data from the Global Biodiversity Information Facility.

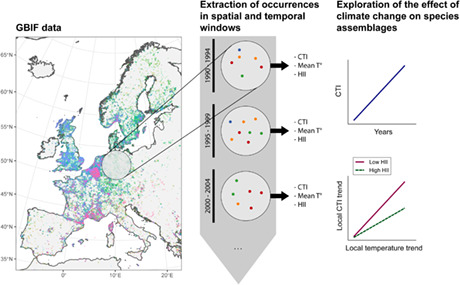

## 1 INTRODUCTION

Climate change is manifested by a strong increase in average global temperatures. Between 1880 and 2012, despite local disparities, the Earth's climate warmed by 0.85°C on average (IPCC, 2015). Among the various global changes currently underway, climate change is emerging as a growing threat to biodiversity (Sala et al., 2000), with effects at all scales of organization, from individual to ecosystem (Bellard et al., 2012). For example, climate change has been shown to alter the phenology and abundance of species (Parmesan, 2006). Changes in climatic conditions also cause shifts in the geographical distribution of species (Chen et al., 2011; Parmesan, 2006). A number of studies have shown that, as temperatures increase, species tend to expand their upper range limits to higher latitudes and altitudes (Barton et al., 2016; Hickling et al., 2005; La Sorte & Thompson, 2007).

At the local scale, changes in species distribution and abundance induce changes in the composition of biological communities, thus creating a temporal turnover of species present at a given site (Jackson & Sax, 2010). Indeed, within an assemblage, increasing temperatures are responsible for the progressive decrease in abundance of relatively cold‐adapted species, until their local extinction. On the contrary, relatively warm‐adapted species increase in abundance and tend to colonize habitats where temperature has become favourable to them. This gradual reshuffling of biological assemblages can be detected by an increase in the community temperature index (CTI, Devictor et al., 2008), a community‐weighted mean of the average temperature to which species are adapted.

To date, several studies have demonstrated this phenomenon of community change in response to climate warming (e.g. Devictor et al., 2012; Martin et al., 2019). However, the vast majority of these have focused on taxa with long‐term systematic monitoring programmes, such as birds and butterflies (Devictor et al., 2008, 2012; Fourcade et al., 2021; Gaüzère et al., 2015; Lehikoinen et al., 2021). In contrast, most taxonomic groups do not have such programmes and as a consequence have not been studied in this aspect yet. Thus, our general knowledge of the modification of species assemblages under climate change currently remains very limited. Moreover, there is evidence that community restructuring in relation to climate generally happens with a debt, that is, there is a lag between the pace of species turnover and the velocity of climate change (Devictor et al., 2012). This debt has been shown to be partly driven by anthropogenic activity that prevents species to shift their range (Fourcade et al., 2021; Gaüzère et al., 2017). However, again, our understanding of the impact of human disturbance on community response to climate change remain limited to the few taxa that benefit from long‐term monitoring schemes.

The geographical distribution of most species on Earth is unknown (Jetz et al., 2012). This lack of knowledge, referred to as the Wallacean shortfall (Lomolino, 2004), is largely caused by the scattering and uneven availability of the many existing data sources. However, the development of public biodiversity databases is helping to fill this gap (Beck et al., 2013). The largest initiative to date is the Global Biodiversity Information Facility (GBIF, www.gbif.org). This international database compiles millions of geo‐referenced and dated species observations, accessible to all and derived from a variety of sources such as museum collections, scientific protocols or citizen science programmes (Edwards, 2004). Public databases have been used extensively to describe spatial patterns of biodiversity (e.g. Gomes et al., 2018), but until very recently had never been exploited to infer temporal changes in community composition. Duchenne et al. (2021) were probably the first to estimate the effect of climate change on the composition of plant species assemblages, and to quantify their climate debt using data from GBIF. It is then interesting to extend this practice to other taxa, especially animals, to understand the impact of global warming on communities in a more comprehensive way and to be able to compare the responses of different groups. However, although GBIF provides information on the distribution of a large number of species, the quality of data is often inferior to that from specific programmes. Therefore, it can be challenging to infer accurately biodiversity trends from such unstructured datasets, and statistical analyses must take into account the uncertain nature of GBIF occurrence data (Beck et al., 2014).

In this study, we used occurrence data from GBIF from Europe and North America to assess the effect of climate change on the assemblages of a variety of animal taxonomic groups that are common study systems in ecology but that do not necessarily benefit from long‐term and large‐scale monitoring programmes (frogs [Anura], bees [Apidae], birds [Aves], bats [Chiroptera], ants [Formicidae], butterflies [Lepidoptera], earthworms [Lumbricidae], rodents [Rodentia] and salamanders [Urodela]). The study had two main objectives. First, we aimed to assess temporal changes in community composition in relation to climate change for a large number of taxa, most of which have never been studied before in the regard, and for a large spatial and temporal scale. Second, another goal of the study was to explore the opportunities and limitations of using data from a public database to detect temporal dynamics of communities.

We attempted to answer the following questions:

Q1: Are the observed changes in community composition compatible with a response to climate change? Here, we estimated change in CTI over time, expecting it to increase because of warming temperatures (Devictor et al., 2008).
Q2: Can community restructuring be related to the recorded local temperature variations? For this, we tested the relationship between the local trend in CTI and the observed local trend in temperature, which should be positive if the increase in CTI is correlated with local temperature change (Devictor et al., 2012; Gaüzère et al., 2017).
Q3: Are communities better adjusted to climate change in natural or anthropogenic environments? This was studied by investigating whether community adjustment to climate change is dependent on anthropogenic disturbance. We hypothesized that species would have a better ability to move in more natural environments, where ecological connectivity is higher (Sonntag & Fourcade, 2022).
Q4: Is community reorganization in response to climate change primarily due to the gain of warm‐adapted species or the loss of cold‐adapted species in assemblages?
Q5: Is the magnitude of the observed community response to climate change influenced by the quantity and quality of the data used? This question was explored by repeating analyses of CTI trend after filtering regions that contain too little data points according to several criteria.

## 2 MATERIALS AND METHODS

### 2.1 Data

#### 2.1.1 Occurrence data

Geo‐referenced observation data were extracted from the GBIF database (www.gbif.org). We chose to study nine animal taxonomic groups, which have long driven attention in ecology owing to their role in ecosystem functioning or because they have served as examples of the current biodiversity decline, and for which we assumed that the amount of data on GBIF was sufficiently large to allow the detection of temporal changes in community composition. For seven out of the nine selected taxa, our study is the first, to our knowledge, that investigates reorganization of communities caused by climate change; at the family level: ants (Formicidae; GBIF, 2022a), earthworms (Lumbricidae; GBIF, 2022b), bees (Apidae; GBIF, 2022c; the genus *Bombus* has already been studied in this regard, see e.g. Fourcade et al., 2019), at the order level: rodents (Rodentia; GBIF, 2022d), bats (Chiroptera; GBIF, 2022e), frogs (Anura; GBIF, 2022f) and salamanders (Urodela; GBIF, 2022g). The last two taxa, the order of butterflies (Lepidoptera; GBIF, 2022h) and the class of birds (Aves; GBIF, 2022i, 2022j), have already been analysed several times with regard to their response to climate change, but not using data from GBIF. Since recent evidence showed that wintering and breeding bird communities respond differently to climate change (Lehikoinen et al., 2021), we separated the birds data into winter (December–January) and summer (March–September). Because some butterfly species can exhibit complex migratory patterns, we kept only occurrences from the breeding season (March–September).

Since GBIF data are prone to a few common and recurrent error types, we performed a first automatic step of occurrence cleaning. For this, we used the R package ‘CoordinateCleaner’ (Zizka et al., 2019) to remove occurrences whose coordinates correspond to the centroid of capitals, countries, GBIF headquarters or known biodiversity institutions, were located in oceans or had equal or exactly 0 latitude and longitude. Moreover, some taxonomic groups have been filtered for species or occurrences. We have removed rats *Rattus norvegicus* and domestic mice *Mus musculus* from the rodent dataset, as well as honeybees *Apis mellifera* from the Apidae data, as these species largely depend on human presence, more than on natural environmental conditions.

Since most of the data from GBIF are posterior to 1990, we decided to conduct this study over the period 1990–2019 (30 years), which should also be long enough to reveal temporal changes in community composition. We divided it into six periods of 5 years, to aggregate more data for calculations and analyses. We used data from all North America (including Central America) and Europe, two continents that differ in climate and habitat types, but remain relatively comparable, being located in the northern hemisphere, and for which the production of GBIF data should be similar.

In opportunistic ecological data, the same individuals may be counted multiple times, and certain areas near roads or cities, and thus easily accessible, may be overrepresented (Mair & Ruete, 2016). Thus, to avoid such bias, we subsampled our dataset by randomly sampling only one occurrence of the same species within cells of a 5 km × 5 km resolution grid, separately for each 5‐year time period. The 5‐km distance was chosen because it is large enough to avoid clumping but small enough not to lose too much data.

To quantify changes in sampling effort and spatial coverage over the study period, we plotted, for each taxon, the number of occurrences and the number of assemblages (defined according to the method outlined in Section 2.2) over the years. We also produced maps of occurrence density in each time period and for each taxon. Because we aimed to assess the suitability of GBIF data to detect changes in community composition, as opposed to standardized protocols, we calculated and plotted the proportion of data attributed to each providing organism.

#### 2.1.2 Climate data and human influence index

We obtained monthly average land temperatures, covering the selected time period (1990–2019), in the form of raster grids with a 0.5° resolution. These climate data are derived from the Climatic Research Unit Gridded Time Series (CRU TS) dataset (Harris et al., 2020) and are produced by interpolating monthly temperature anomalies, calculated from weather station observations. We produced rasters of mean temperature for each of the six 5‐year periods, using March–September months (for breeding birds and butterflies), December–January months (wintering birds) or all 12 months (for all other taxa).

To assess the effect of human disturbance on the ability of communities to track climate change, we used a human influence index (Sanderson et al., 2002). This index was created by assembling nine layers of data that provide information on the anthropization of the environment, such as human population density, land use or the presence of infrastructure. The global, 30 arc‐sec resolution, human influence index grid for the period 1995–2004 was obtained from the Last of the Wild collection of the SEDAC (Socio‐Economic Data and Applications Center) database.

### 2.2 Sliding windows

**FIGURE 1:**
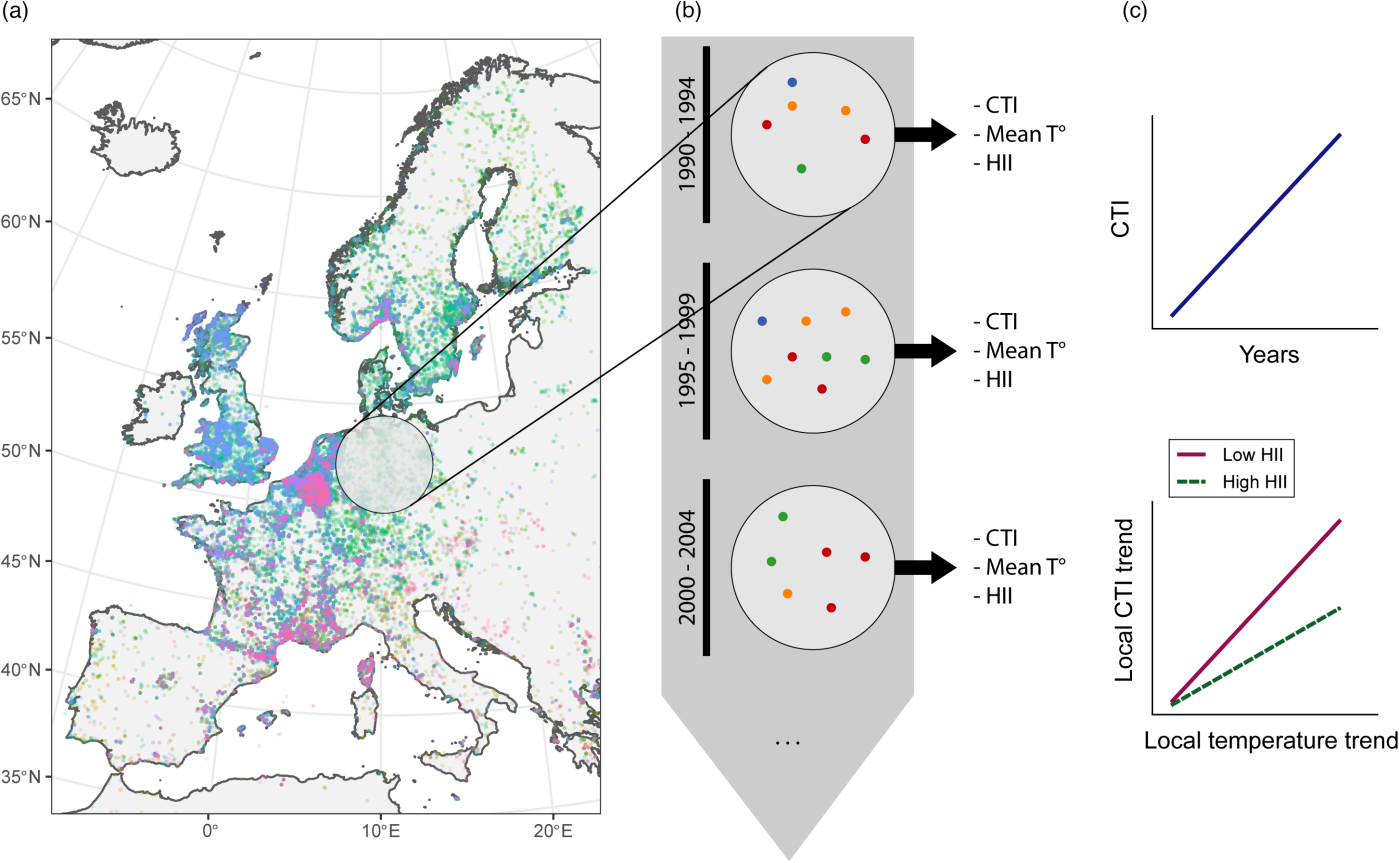
Summary diagram of the methods. (a) Portion of the study area showing as an example the ant dataset in Europe (species in different colours) with a sliding window. The real sliding windows have a 200‐km radius and are interspaced by 1° (b) zooming in on a sliding window: For each 5‐year period, occurrences of different species (figured by different colours) were used to calculate community temperature index (CTI), while mean local temperature and human influence index (HII) were also extracted. (c) Expected relationships between CTI and time (top), and local CTI trend and local temperature trend in interaction with human influence index (bottom).

To create species assemblages with as many occurrences as possible, we followed a sliding window approach inspired by Gaüzère et al. (2015). Around the centroids of 1° resolution grid cells, we created buffers with a radius of 200 km and extracted all occurrences (Figure 1a), which we will refer to as sliding windows from now on. This allows to consider more data within each community while not relying on subjective boundaries for delineating assemblages. Within each sliding window that contained records from at least two species (5997 windows in total), we calculated community indices (see Section 2.3) and the mean values of human influence index and temperature (Figure 1b). We also estimated the observed local temporal trends in temperature over the study period. To do this, within each sliding window, we performed linear regressions of temperature against median years of the 5‐year periods.

### 2.3 Community and species temperature indices

For each taxonomic group, we extracted temperature at each occurrence from the temperature grids, considering both its location and its year of observation. A temperature index was computed for each species (species temperature index [STI]) as the mean temperature recorded across all cleaned and spatially subsampled occurrences. This should correspond to the average temperature experienced by species within their range and therefore be indicative of their thermal adaptation (Devictor et al., 2008).

Then, within each sliding window and for each 5‐year period, a CTI was calculated as the arithmetic mean of the STI values for each species present in the assemblage (Figure 1b). The use of GBIF data did not allow for the calculation of an abundance‐weighted CTI, but only a presence‐absence CTI based on the identity of species recorded in a sliding window. Temporal trends in CTI, reflecting community rearrangement in response to climate change, are expected to correlate with temporal trends in temperature variation at local scale, and to be positive because of warming temperatures (Figure 1c). In theory, a perfect adjustment of communities to temperature change would lead to a regression coefficient of 1 between CTI trend and temperature trend. A weaker or non‐existent relationship means that the observed changes in community mean temperature do not correspond well to temporal changes in local temperature, which could potentially be caused by human disturbance (Figure 1c).

### 2.4 Temporal community change

To assess changes in CTI over time (Q1), CTI—calculated for each sliding window and each time period—was modelled (separately for each taxon) as a function of the median year of the 5‐year periods, incorporated as a continuous fixed‐effect variable, using linear mixed models. We also considered the fixed effects of the continent, as well as mean temperature calculated within each window. We included sliding window identity as a random intercept, and allowed random slopes of time depending on sliding window. We incorporated as an additional random intercept the identity of the largest ecoregion contained within each sliding window, extracted from the WWF's Terrestrial Ecoregions of the World (Olson et al., 2001). We found these model settings to be sufficient to control for spatial autocorrelation, as verified by spline correlograms of model residuals, while explicitly modelling spatial covariance structure was almost computationally intractable. Models were weighted by the logarithm (to limit skewness) of the number of occurrences in each sliding window and each year, therefore controlling for variation in sampling effort and sliding windows' area (which is reduced in coastal regions). We reported for each taxonomic group the estimate and 95% confidence interval of the temporal trend in CTI.

To determine whether the temporal change in CTI was related to observed temperature variations (Q2), we modelled the local temporal trend in CTI as a function of the local temporal trend in temperature, in interaction with average temperature because this relationship may differ depending on baseline climatic conditions. Local CTI temporal trends were extracted from the random slopes of the relationship between CTI and time. We also aimed to investigate whether the ability of communities to track climate change depended on human disturbance (Q3). For this reason, we included in the model the interaction between local temporal trend in temperature and the human influence index. In addition, the model included continent as an additional covariable, as well as ecoregion as a random intercept. In addition, it was also weighted by the log‐transformed number of occurrences used to produce CTI estimates in each sliding window. Here, we extracted the estimate and 95% confidence interval of the relationship between the temporal trend in CTI and the temporal trend in local temperature for three contrasted values of the human influence index, corresponding to the 25th percentile, median and 75th percentile of observed values.

For each of the models described above, we calculated meta‐analytic means and confidence intervals using the *rma* function in the metafor R package (Viechtbauer, 2010), to estimate the average community change over time for all taxa studied, as well as the average relationships between CTI and temperature trends (for low, medium and high values of human influence index).

### 2.5 Species turnover

To understand the species‐level processes that lead to CTI change over time (Q4), we assessed the respective contribution of species to changes in CTI. An increase in CTI is due to either the loss of cold‐adapted species or the gain of warm‐adapted species. In each time interval [*t* – *t*‐1] and for each sliding window, we identified the species that were gained and those that were lost. Note that, given the nature of data, we cannot be certain that these species were new colonizers or truly went extinct. For each of these species, we then calculated a relative STI (rSTI, following Fourcade et al., 2021) as the difference between the species' STI and the CTI at year *t* − 1 of the interval. A species with rSTI >0, that is, STI greater than the CTI of the assemblage at year *t* − 1, is warm adapted in its local environment. Conversely, a species with rSTI < 0 can be considered cold adapted. For each taxonomic group, we calculated the average rSTI of lost and gained species. Comparing rSTI between species gained and species lost indicates whether the change in CTI is mostly due to the gain of warm‐adapted species or the loss of cool‐adapted species.

### 2.6 Effect of data quantity

Finally, we assessed the effect of the amount of data on the estimated response of different taxa to climate change (Q5). For this purpose, we reanalysed the temporal change in CTI after applying additional filtering criteria to keep only sliding windows that contained a certain minimum number of occurrences across all species (50, 75, 100, 125 or 150) or that contained data for a minimum of two, three, four or five 5‐year time periods. We compared the overall slope of the relationship between CTI and time with the slopes estimated from the full dataset, using Pearson correlation tests. We also extracted, from the random slopes of the model, the local CTI trends in each sliding window, and calculated the absolute difference in CTI trends between the filtered and full datasets. We tested the effects of taxon, the minimum number of occurrences and the minimum number of 5‐year periods, as well as their three‐way interaction, using a linear mixed model with the identity of sliding windows as a random intercept. We graphically explored the results by plotting the predicted outcome across the range of observed values of the explanatory variables.

## 3 RESULTS

### 3.1 Data quantity and sources

**FIGURE 2:**
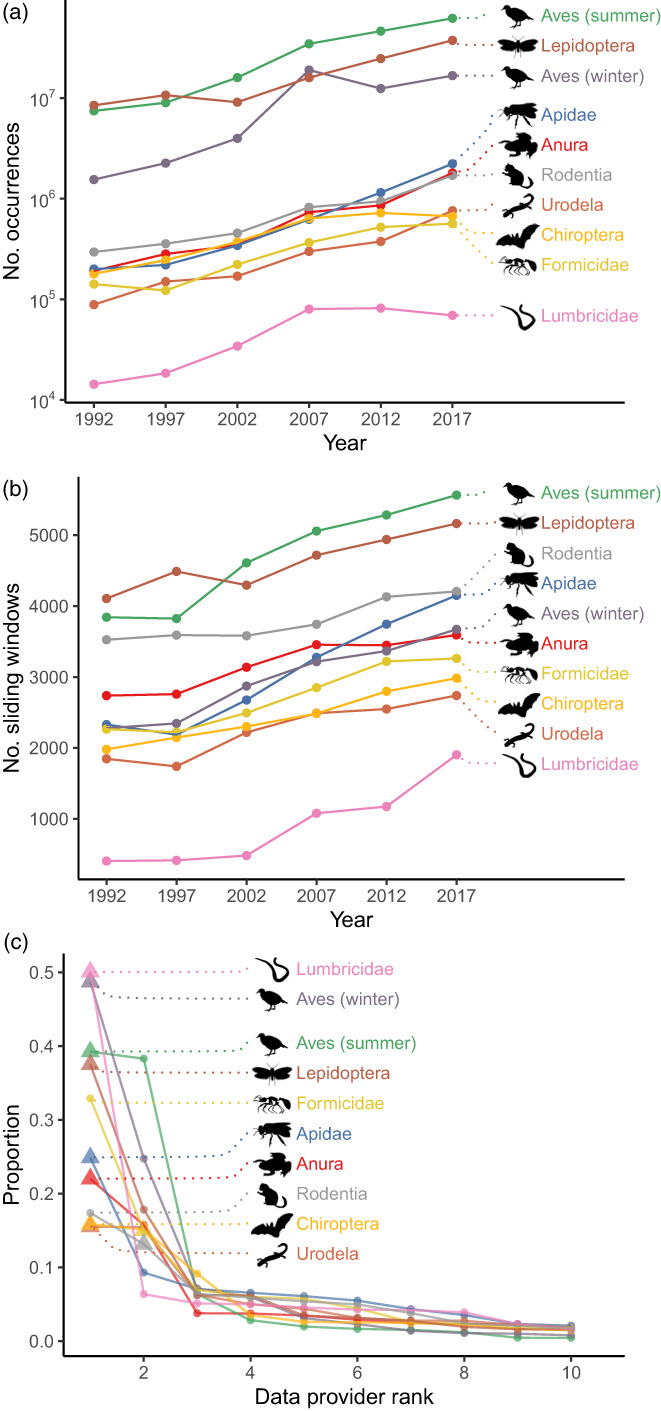
Characteristics of Global Biodiversity Information Facility (GBIF) data extracted in this study. (a, b) Temporal change in sampling effort after occurrence cleaning and spatial subsampling (number of occurrences per year, a) and spatial coverage (number of sliding windows containing data, b). (c) Heterogeneity of data sources, shown as the proportion of data per providing organizations in decreasing rank order. For graphical visualization, only the 10 first ranks were kept for each taxon. Unknown data providers are represented as triangles.

In total, our data contained 24,376 butterfly species, 2724 summer bird species, 2352 wintering bird species, 2283 ant species, 1307 bee species, 753 frog species, 560 rodent species, 481 urodele species, 270 bat species and 87 earthworm species. The number of occurrences increased exponentially over the study period for all taxa studied, except earthworms for which it reached a plateau in the last 5‐year periods (Figure 2a). The number of sliding windows containing occurrences, that is, the number of species assemblages that were considered in analyses, also increased over the years (Figure 2b). Occurrence density was consistently higher in Western/Northern Europe than in any other region. There was no strong change in spatial coverage overall, but we observed a notable increase in data quantity in North America during the last time periods (Supporting Information, Figure S1).

Biodiversity data from public databases are characterized by the aggregation of a multitude of different sources. For all taxa but rodents and ants, the largest ‘provider’ was in fact unknown, that is, this information is missing from the GBIF database. It represents ca. 50% of all occurrence data for earthworms and wintering birds (Figure 2c). Generally, a few data providers represented a large proportion of the available occurrences, especially for birds where the top‐ranked organization (Artdatabanken) provided 38% (summer birds) and 25% (winter birds) of all data. Salamanders had the most equitable distribution of sources, with 20 organisms providing more than 1% of the total amount of data (15% for the first one, Figure 2c).

### 3.2 Community dynamics

**FIGURE 3:**
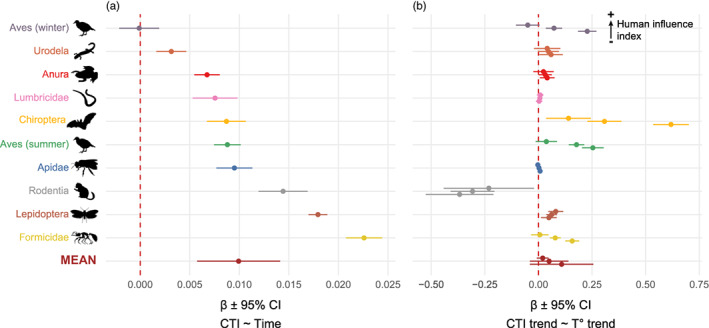
Community response to climate change for each taxon. Slopes and 95% confidence intervals of the relationship between (a) community temperature index (CTI) and time (i.e. 5‐year periods), (b) local CTI trend and local temperature trend, shown for three levels of human influence index (25th percentile, median, and 75th percentile of values, from bottom to top). The brown dots at the bottom represent the meta‐analytical mean responses across all taxa.

During the study period (1990–2019), we observed overall a positive temporal change in CTI (*β*_meta_ = 0.0099°C year^−1^ [CI_95%_: 0.0057–0.0141]), signifying that communities were increasingly composed of species relatively adapted to elevated temperatures (Figure 3a), although with spatial variation (Figure S2). CTI increased very significantly for 9 out of the 10 taxonomic groups studied: ants (*β* = 0.0226 [CI_95%_: 0.0208–0.0244]), butterflies (*β* = 0.0179 [CI_95%_: 0.0170–0.0189]), rodents (*β* = 0.0144 [CI_95%_: 0.0119–0.0169]), bees (*β* = 0.0095 [CI_95%_: 0.0077–0.0133]), summer birds (*β* = 0.0088 [CI_95%_: 0.0074–0.0101]), bats (*β* = 0.0087 [CI_95%_: 0.0067–0.0107]), earthworms (*β* = 0.0076 [CI_95%_: 0.0053–0.0098]), frogs (*β* = 0.0067 [CI_95%_: 0.0055–0.0080]) and salamanders (*β* = 0.0031 [CI_95%_: 0.0016–0.0046]). However, winter birds did not experience a significant variation in CTI over the study period (*β* = −0.0001 [CI_95%_: −0.0021–0.0019]; Figure 3a).

At the scale of sliding windows, it was less clear whether the temporal trend in CTI was positively correlated with the trend in temperature, since it was overall positive as expected but with wide confidence intervals that include 0 (Figure 3b, *β*_meta_ = 0.0200–0.1083 [°C year^−1^]_CTI_/[°C year^−1^]_temperature_ depending on human influence index). This relationship was significantly positive, for median value of human influence index, for bats (*β* = 0.3081 [CI_95%_: 0.2289–0.3873]), summer birds (*β* = 0.1778 [CI_95%_: 0.1416–0.2140]), ants (*β* = 0.0786 [CI_95%_: 0.0534–0.1038]), winter birds (*β* = 0.0727 [CI_95%_: 0.0347–0.1107]), butterflies (*β* = 0.0617 [CI_95%_: 0.0364–0.0869]) and frogs (*β* = 0.0322 [CI_95%_: 0.0019–0.0625]). There was no significant link between CTI and temperature trends for salamanders (*β* = 0.0468 [CI_95%_: −0.0023–0.0959]), earthworms (*β* = 0.0068 [CI_95%_: −0.0041–0.0177]) and bees (*β* = 0. 0028 [CI_95%_: −0.0002–0.0058]). At median value of human influence index, we found a negative relationship for rodents (*β* = −0.3074 [CI_95%_: −0.4096 to −0.2052]) only (Figure 3b).

The interaction between temperature trends and human influence index was significant for bees, birds (winter & summer), bats and ants only (Table S1b). For all these taxa, the relationship between CTI and temperature trends was weaker with high values of the human influence index (Figure 3b), meaning that these assemblages did not track climate change as efficiently in anthropized areas.

### 3.3 Contribution of species to community reorganization

**FIGURE 4:**
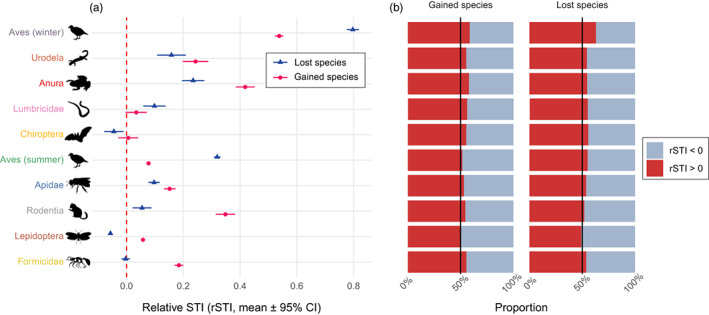
(a) Mean relative species temperature index (rSTI) of species lost (dark blue triangles) and gained (pink dots) between each time period. (b) Proportion of gained and lost species that had positive (red) or negative (blue) rSTI. For a given species, its rSTI represents the difference between the STI and the community temperature index of the assemblage it colonizes or from which it is extirpated, therefore reflecting whether it is locally warm (rSTI > 0) or cold (rSTI < 0) adapted.

We found that in most taxa the mean rSTI was >0 for both lost and gained species (Figure 4a), which means that the species that appeared or disappeared from the data between two time periods were mainly those adapted to warmer climates than species previously present in the assemblages. In bats and butterflies, however, lost species had a mean rSTI < 0. In birds (both winter and summer), lost species had a significantly higher rSTI than gained species, contrary to all other taxa where gained species had higher mean rSTI (except earthworms for which confidence intervals of rSTIs of gained and lost species overlapped).

In all taxa, the majority of species gained between two time periods had positive rSTI. Interestingly, we also found that >50% of species lost between two time periods had positive rSTI, expect for butterflies where species with rSTI < 0 accounted for 51% of lost species (Figure 4b).

### 3.4 Effect of data quantity on observed community response to climate change

**FIGURE 5:**
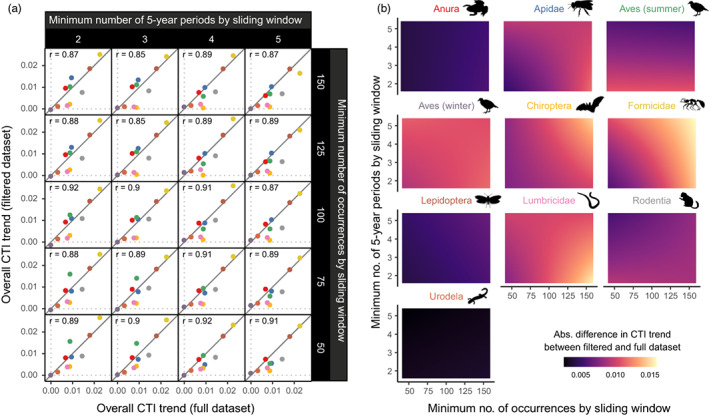
Effect of data filtering on the estimated temporal trend in community temperature index (CTI). (a) Correlation (scatterplots + Pearson's correlation coefficient) between taxon‐level CTI trends estimated from filtered datasets (*y*‐axis) and CTI trends estimated from the whole datasets (*x*‐axis), for different levels of data filtering applied at the level of sliding windows (columns: minimum of 2–5 time periods; rows: minimum of 50–150 occurrences). The grey line represents the 1:1 line. (b) Predicted absolute difference in CTI trends estimated from filtered or full datasets, as a function of the minimum number of 5‐year time periods (*y*‐axis) and occurrences (*x*‐axis) by sliding window, for each taxon.

We found a strong agreement in the overall estimates of CTI temporal trend whatever the level of data filtering; in all cases, we observed a correlation of ca. 0.9 between estimates obtained from the full datasets and from the datasets filtered for a certain minimum of time periods or occurrences by sliding window (Figure 5a).

At the scale of each sliding window, we observed that, although estimates of CTI trends could vary with data filtering, the difference with the estimates obtained from the whole dataset remained overall centred around zero (Figure S3). Still, for bats and even more for earthworms, the estimated temporal trends in CTI tended to be lower when we used only the sliding windows that contain a minimum number of years or occurrences (Figure S3).

The three‐way interaction between taxon, minimum number of years and minimum number of occurrences had a strongly significant effect on the absolute difference between estimates (*χ* ^2^ = 265.61, *p* < 0.001), showing that both types of data filtering had different interacting effects depending on the taxonomic groups considered. Indeed, while estimates of CTI trends were on average extremely stable for frogs, butterflies and salamanders, for ants and bats the deviation was larger when both filtering criteria were stricter (Figure 5b). In earthworms, the largest differences were found when we selected only sliding windows with >150 occurrences, while the number of time periods seems less important. The other taxa showed deviations that were harder to attribute to one or the other filtering criterion (Figure 5b).

## 4 DISCUSSION

Using data from the GBIF, we characterized the effect of climate change on the assemblages of a variety of animal taxonomic groups, the majority of which had never been studied before with regard to their response to climate change. For all but one taxa, we observed a significant increase in CTI over the period 1990–2019 at the scale of Europe and North America, revealing that communities are generally restructured as a response to climate change. At the local scale, we found that community composition changes following temperature variation for most of the taxa studied (only one showed a clear negative response), although with a lag (i.e. relationship <1), suggesting that communities are experiencing a climatic debt. This climatic debt appeared to be greater in highly anthropized regions. In addition, we found that the increase in CTI is mostly caused by the arrival of warm‐adapted species and not the loss of cold‐adapted species, for all taxonomic groups except butterflies.

All of the nine different taxonomic groups we analysed showed evidence of a thermophilization in response to climate change (Figure 3a). Among the taxa we selected, this pattern had already been shown in birds and butterflies (e.g. Devictor et al., 2012). Recently, Lehikoinen et al. (2021), who compared the response of wintering and breeding bird communities to climate change at the scale of Europe and North America, found that wintering communities are reshaped more rapidly than breeding communities. Here, surprisingly, we found instead that, although summer bird communities showed a significant ‘warming’, there was no detectable increase in CTI for wintering birds' communities during the study period. Although it seems to contradict previous evidence, it appears that when we restrict data to the same countries as in Lehikoinen et al. (2021), we do in fact observe a clear increase in CTI over time (*β* = 0.01347 [0.0095–0.0174]). This demonstrates that such assessment of community change can be highly dependent on the input data, including its spatial and temporal coverage. This reinforces the need to find additional sources of information to complement the existing standardized protocol, such as the GBIF as we explore here (Beck et al., 2013).

A positive relationship between estimated local CTI trends and observed local temperature trends was found in 6 out of 10 groups (Figure 3b), indicating that most communities are indeed structured by climate. Still, in several taxa, despite evidence for an overall thermophilization of community over time, local variations of the CTI were only weakly correlated with those of temperature. Several hypotheses can explain this apparent discrepancy. First of all, community dynamics of many animal groups may depend in part on microclimatic variation that is not well captured by the macroclimate variables we use. This can be the reason why earthworms, which are soil dwellers (Lavelle, 1988), do not show a significantly positive relationship between local CTI and temperature trends. Similarly, many amphibians depend on water ecosystems for their reproduction (Wells, 2007). Therefore, we expect water‐dependant frog and salamander species to track changes in freshwater temperature, which may differ from air temperature. Rodents, for which we observed a negative relationship between CTI and temperature trends, present an interesting case that is difficult to explain. Perhaps their particular lifestyle makes them live in microclimates that change opposite to the observed macroclimate. Second, we characterized community change and temperature trends at the scale of 200‐km‐radius sliding windows. In doing so, we assumed ‘local’ temperature trends to be the average change in temperature across >125,000 km^2^. This may very well be too large to represent accurately the climate change that communities are responding to. Among the other studies that applied a sliding window approach, Gaüzère et al. (2017) analysed changes in community composition in 80‐km‐radius circles as a way to incorporate at least 20 monitoring sites. Here, we relied instead on unstructured occurrence data that are prone to biases and error (Beck et al., 2014). Therefore, we chose to analyse large‐scale assemblages that are certainly not communities in the sense of a group interacting species, but which size seemed reasonable to describe local groups of species while avoiding putting too much weight on unavoidable biases in the data. The optimal scale at which such data must be analysed remain an open question, though.

Importantly, for all the taxa studied, the relationship between local CTI trend and temperature trend was less than 1 (Figure 3b). This means that there is a lag between changes in community composition and climate change, also known as climate debt (Devictor et al., 2012). This lag may be an indicator of the difficulty of communities to keep up with rapid changes in climate, as it is often the case in terrestrial habitats, but less so for marine species (Lenoir et al., 2020). The intrinsic dispersal abilities of species can contribute to this lag, but we also know for sure that habitat availability (Mair et al., 2014) and habitat fragmentation (Fourcade et al., 2021) play a role in preventing climate‐driven range shifts. In this regard, we found that communities tracked better climate change in natural habitats as opposed to areas disturbed by human activity. Here, the human influence index we used is an aggregation of several types of human footprints (Sanderson et al., 2002) and cannot be used to discriminate the exact factors that prevent community reorganization. Among the nine animal taxa we studied, it is likely that different types of disturbance act differently to prevent climate tracking. For example, amphibian range shifts may be impaired by the pollution or loss of water ponds (Araújo et al., 2006), while insect taxa are known to be strongly impacted by habitat fragmentation (Fourcade et al., 2021). In birds, it has been demonstrated that habitat diversity and the naturalness of the landscape influenced their climatic debt (Gaüzère et al., 2017).

It must be noted that this apparent lag may not necessarily mean that species are at risk of extinction because of climate change. Changes in community composition, such as those revealed by an increase in CTI, reflect shifts in the distribution of species that track their climatic niche in space (Devictor et al., 2008). Therefore, lack of distribution shifts could on the opposite be an indicator of the ability of species to adapt to new thermal conditions, making range shifts unnecessary even when climate is changing. Indeed, some species may exhibit phenological shifts, or have already adapted to warmer conditions (Parmesan, 2006). In this case, maladaptation to climate change cannot be concluded simply from a mismatch between CTI and temperature trends. In addition, it is essential to remember that, due to the nature of GBIF data, we chose to focus our work on the temporal trends in presence‐only CTI, which represent only the turnover in species composition (and thus distribution shifts of species), ignoring fluctuations in abundances. A large part of the changes occurring in communities as a response to climate change may thus remain undiscernible in our analyses, such as a decline in cold‐adapted species (which can in some cases precede their extinction) or an increase in the abundance of warm‐adapted species. A better understanding of these processes could be obtained if GBIF occurrence density could be interpreted in terms of species' abundance. Still, it is remarkable that a temporal increase in presence‐only CTI was detected in almost all cases here; this suggests that spatial climate tracking is a widespread response to climate change across various terrestrial taxa.

By analysing turnover processes at the species level, we found that for 7 out of 10 taxonomic groups, the average relative STI of species gained between two time periods is higher than that of species lost (Figure 4), which is expected if communities are restructured in response to climate change. However, we expected to find a negative rSTI for lost species if the loss cold‐adapted species contributed to the increase in CTI, or alternatively rSTI_lost_ ≈ 0 if the arrival of warm‐adapted species in communities was the primary driver of increasing in CTI over time. Here, instead, the average rSTI of lost species was positive for seven taxonomic groups, suggesting that the species lost from communities over time are also mostly warm‐adapted species. The result is confirmed by the fact that the majority of both gained and lost species showed a higher STI than the CTI of their community (rSTI > 0). We interpret this result by the fact that newly gained species, if they are colonizing as they track climate change, are located at their climate niche boundary and are thus naturally more sensitive to local extinction risks. Thus, warm‐adapted species that colonize sites where temperature has become favourable to them could also be species that are easily lost from communities in the next years, before they can effectively establish in the long term. Interestingly, we observed a more expected outcome for butterflies, where species that are lost tend to be cold‐adapted relative to the communities from which they are extirpated (rSTI < 0). For this taxon at least, it appears thus that community turnover is driven by both the arrival of warm‐adapted species and the loss of cold‐adapted species, which is consistent with species range shifts caused by increasing temperatures.

Finally, we found that filtering data to keep only the regions that contain a certain number of data points influenced relatively little the estimated temporal trends in CTI at the level of each taxon (Figure 5), that is, our main results are robust to the inclusion or not of undersampled area. This is partly due to the fact that we already weighted our statistical models by the density of occurrences in each sliding window, therefore giving less weight to undersampled areas. There is a clear spatial variation in the amount of data available (Figure S1), Western Europe being generally overrepresented. It is also noticeable that Central America (included here with North America) appears relatively undersampled, despite the fact that it is one of the main biodiversity hotspots worldwide (Myers et al., 2000). Increasing the spatial coverage of robust biodiversity data may help deciphering the variation of species and community response to climate change across biomes and taxa. In this regard, the database GBIF may play an important role by allowing researchers to rely on unstructured datasets, providing that such data are properly cleaned, filtered and analysed. Local‐scale trends in CTI are more difficult to estimate, and proved more sensitive to the process of data filtering. Here, the temporal completeness of data appears as an important factor, at least for some taxa, suggesting that long‐term monitoring programmes are essential for estimating changes in community composition, but also that assessments of biodiversity response to climate change will improve over time as more and more occurrence data are included in the GBIF.

In conclusion, we observed generally that climate change is causing major shifts in species assemblages for many different taxa, characterized by an increase in the proportion of warm‐adapted species at the expense of cold‐adapted species. This reorganization of communities appears to be mostly the result of the colonization of new warm‐adapted species, although there may be hidden trends in species' abundance which also contribute to changes within communities. Our study cannot strictly prove that GBIF occurrence data are appropriate for such temporal assessment, but the fact that we consistently observed responses that are expected in a climate change context strongly suggests that the effects we detected are real. Additional taxa for which sufficient GBIF data exist could then be analysed with the same methods, thus improving knowledge of the impact of climate change on biodiversity. Moreover, the framework we employed here could be extended for the study of other long‐term and large‐scale processes such as biotic homogenization (McKinney & Lockwood, 1999). Another, applied, conclusion we gained from our study was that communities are lagging behind climate change, and that this lag is partly caused by human disturbance. Thus, conservation solutions, such as the deployment of ecological corridors to promote the movement of species to environments that satisfy their thermal preferences, would be necessary (Littlefield et al., 2019; Sonntag & Fourcade, 2022). We demonstrated here that these issues may exist for a large range of terrestrial animals, and we introduced a framework that can be used in a broader context, opening new opportunities for global change research.

## AUTHOR CONTRIBUTIONS

Armelle Lajeunesse: analysed the data and wrote the original draft of the manuscript. Yoan Fourcade: designed the ideas and methodology, and analysed the data. All authors contributed to the final version of the manuscript and gave final approval for publication.

## CONFLICT OF INTEREST

The authors declare that they have no conflict of interest.

## Supporting information


Data S1.

## Data Availability

All the data that were used in the manuscript are freely available online. Species occurrence data were obtained from GBIF (GBIF, 2022a, DOI: https://doi.org/10.15468/dl.ad98e2; GBIF, 2022b, DOI: https://doi.org/10.15468/dl.scb6hm; GBIF, 2022c, DOI: https://doi.org/10.15468/dl.6uhd6h; GBIF, 2022d, DOI: https://doi.org/10.15468/dl.bhueq2; GBIF, 2022e, DOI: https://doi.org/10.15468/dl.v5nnww; GBIF, 2022f, DOI: https://doi.org/10.15468/dl.d3w4td; GBIF, 2022g, DOI: https://doi.org/10.15468/dl.gxq5nb; GBIF, 2022h, DOI: https://doi.org/10.15468/dl.bwkbth; GBIF, 2022i, DOI: https://doi.org/10.15468/dl.s7ng4g; GBIF, 2022j, DOI: https://doi.org/10.15468/dl.5pask5). Climate data and human influence index were extracted in each sliding window and made available in the Figshare repository (Fourcade & Lajeunesse, 2022, DOI: https://doi.org/10.6084/m9.figshare.21632732.v2). R scripts are available as a GitHub repository archived in Zenodo (Fourcade, 2022, DOI: https://doi.org/10.5281/zenodo.7343539).
