# Supplementary material for: Temporal analysis of GBIF data reveals the restructuring of communities following climate change
Source: J Anim Ecol. 2022 Dec 7;92(2):391–402. doi: 10.1111/1365-2656.13854 (PMC10107267; doi:10.1111/1365-2656.13854)

Temporal analysis of GBIF data reveals the restructuring of communities following climate change

Lajeunesse, A. & Fourcade, Y.
Univ Paris Est Creteil, Sorbonne Université, Université Paris-Cité, CNRS, IRD, INRAE, Institut d'écologie et des sciences de l'environnement, IEES, F-94010 Créteil, France

Journal of Animal Ecology

Supporting information

Table S1: Summary of the fixed effects of statistical models of (a) CTI as a function of time (5-year periods), (b) local CTI trend as a function of local temperature trend, in interaction with the human influence index (HII). Full models also include random factors, and are weighted by the number of occurrence in each sliding window.

(a) CTI ~ Time

Taxon	Variable	β	SE	P value	Lower $CI_{95\%}$	Upper $CI_{95\%}$
Anura	Intercept	6.5092	0.2904	0.0000	5.9399	7.0784
	Time	0.0067	0.0007	0.0000	0.0055	0.0080
	Mean temperature	0.4163	0.0066	0.0000	0.4035	0.4292
	Continent [N. Am.][a]	2.5611	0.3307	0.0000	1.9130	3.2092
	R^2_m	0.7459				
	R^2_c	0.9253				
Apidae	Intercept	5.5427	0.2904	0.0000	4.9734	6.1119
	Time	0.0095	0.0009	0.0000	0.0077	0.0113
	Mean temperature	0.4215	0.0065	0.0000	0.4088	0.4343
	Continent [N. Am.][a]	0.9672	0.2679	0.0003	0.4421	1.4922
	R^2_m	0.5702				
	R^2_c	0.8574				
Aves (summer)	Intercept	10.9538	0.1957	0.0000	10.5702	11.3374
	Time	0.0088	0.0007	0.0000	0.0074	0.0101
	Mean temperature	0.2607	0.0045	0.0000	0.2519	0.2696
	Continent [N. Am.][a]	0.7667	0.1264	0.0000	0.5189	1.0144
	R^2_m	0.3979				
	R^2_c	0.7429				
Aves (winter)	Intercept	1.7139	0.3765	0.0000	0.9759	2.4519
	Time	-0.0001	0.0010	0.9229	-0.0021	0.0019
	Mean temperature	0.3007	0.0049	0.0000	0.2911	0.3103
	Continent [N. Am.][a]	4.5050	0.4262	0.0000	3.6697	5.3404
	R^2_m	0.6108				
	R^2_c	0.8752				
Chiroptera	Intercept	7.6509	0.5732	0.0000	6.5275	8.7744
	Time	0.0087	0.0010	0.0000	0.0067	0.0107
	Mean temperature	0.3762	0.0078	0.0000	0.3609	0.3914
	Continent [N. Am.][a]	2.8626	0.6462	0.0000	1.5960	4.1291
	R^2_m	0.4036				
	R^2_c	0.9338				
Formicidae	Intercept	4.6507	0.2669	0.0000	4.1276	5.1738
	Time	0.0226	0.0009	0.0000	0.0208	0.0244
	Mean temperature	0.5562	0.0062	0.0000	0.5441	0.5682
	Continent [N. Am.][a]	1.7981	0.3031	0.0000	1.2041	2.3921
	R^2_m	0.8116				
	R^2_c	0.9090				
Lepidoptera	Intercept	7.2681	0.2880	0.0000	6.7037	7.8326
	Time	0.0179	0.0005	0.0000	0.0170	0.0189
	Mean temperature	0.4884	0.0055	0.0000	0.4776	0.4992
	Continent [N. Am.][a]	0.2413	0.1858	0.1948	-0.1229	0.6056
	R^2_m	0.5588				
	R^2_c	0.9250				
Lumbricidae	Intercept	9.0129	0.2070	0.0000	8.6072	9.4186
	Time	0.0076	0.0012	0.0000	0.0053	0.0098
	Mean temperature	0.1053	0.0060	0.0000	0.0935	0.1171
	Continent [N. Am.][a]	-1.1642	0.2314	0.0000	-1.6178	-0.7106
	R^2_m	0.2274				
	R^2_c	0.7470				
Rodentia	Intercept	4.5081	0.2684	0.0000	3.9820	5.0341
	Time	0.0144	0.0013	0.0000	0.0119	0.0169
	Mean temperature	0.5263	0.0068	0.0000	0.5129	0.5397
	Continent [N. Am.][a]	0.3244	0.2446	0.1850	-0.1550	0.8038
	R^2_m	0.7148				
	R^2_c	0.8985				
Urodela	Intercept	6.3109	0.3079	0.0000	5.7075	6.9143
	Time	0.0031	0.0008	0.0001	0.0016	0.0046
	Mean temperature	0.4452	0.0103	0.0000	0.4250	0.4654
	Continent [N. Am.][a]	1.7073	0.3402	0.0000	1.0404	2.3741
	R^2_m	0.7026				
	R^2_c	0.9399				

[a] Europe taken as reference

(b) CTI trend ~ T° trend

Taxon	Variable	β	SE	P value	Lower CI95%	Upper CI95%
Anura	Intercept	0.0101	0.0012	0.0000	0.0077	0.0125
	T° trend	-0.0215	0.0256	0.4004	-0.0717	0.0287
	Mean temperature	0.0001	0.0001	0.2011	0.0000	0.0002
	HII	-0.0003	0.0001	0.0000	-0.0004	-0.0001
	Continent [N. Am.][a]	-0.0013	0.0010	0.1912	-0.0033	0.0006
	T° trend : Mean temperature	0.0078	0.0021	0.0002	0.0038	0.0119
	T° trend : HII	-0.0011	0.0019	0.5480	-0.0048	0.0025
	R^2_m	0.0177				
	R^2_c	0.0872				
Apidae	Intercept	0.0095	0.0001	0.0000	0.0092	0.0097
	T° trend	0.0064	0.0026	0.0138	0.0014	0.0114
	Mean temperature	0.0000	0.0000	0.0061	0.0000	0.0000
	HII	0.0000	0.0000	0.1487	0.0000	0.0000
	Continent [N. Am.][a]	0.0000	0.0001	0.6734	-0.0002	0.0001
	T° trend : Mean temperature	0.0006	0.0002	0.0141	0.0001	0.0011
	T° trend : HII	-0.0006	0.0002	0.0210	-0.0010	-0.0001
	R^2_m	0.0020				
	R^2_c	0.0056				
Aves (summer)	Intercept	0.0033	0.0013	0.0117	0.0007	0.0058
	T° trend	0.1958	0.0281	0.0000	0.1408	0.2507
	Mean temperature	-0.0005	0.0001	0.0000	-0.0007	-0.0003
	HII	0.0003	0.0001	0.0000	0.0002	0.0005
	Continent [N. Am.][a]	0.0037	0.0009	0.0001	0.0019	0.0056
	T° trend : Mean temperature	0.0101	0.0023	0.0000	0.0056	0.0146
	T° trend : HII	-0.0115	0.0020	0.0000	-0.0153	-0.0076
	R^2_m	0.0129				
	R^2_c	0.0295				
Aves (winter)	Intercept	-0.0096	0.0020	0.0000	-0.0136	-0.0056
	T° trend	0.4228	0.0393	0.0000	0.3457	0.4999
	Mean temperature	-0.0005	0.0001	0.0000	-0.0006	-0.0003
	HII	0.0004	0.0001	0.0000	0.0002	0.0006
	Continent [N. Am.][a]	0.0002	0.0012	0.8696	-0.0022	0.0026
	T° trend : Mean temperature	0.0219	0.0020	0.0000	0.0179	0.0258
	T° trend : HII	-0.0189	0.0023	0.0000	-0.0234	-0.0145
	R^2_m	0.0174				
	R^2_c	0.0353				
Chiroptera	Intercept	0.0080	0.0033	0.0147	0.0016	0.0144
	T° trend	0.2179	0.0756	0.0041	0.0696	0.3661
	Mean temperature	-0.0022	0.0002	0.0000	-0.0026	-0.0019
	HII	0.0008	0.0002	0.0000	0.0005	0.0011
	Continent [N. Am.][a]	0.0021	0.0022	0.3459	-0.0022	0.0063
	T° trend : Mean temperature	0.0740	0.0047	0.0000	0.0648	0.0832
	T° trend : HII	-0.0352	0.0045	0.0000	-0.0440	-0.0265
	R^2_m	0.0762				
	R^2_c	0.1457				
Formicidae	Intercept	0.0191	0.0009	0.0000	0.0173	0.0210
	T° trend	0.0896	0.0204	0.0000	0.0497	0.1295
	Mean temperature	-0.0005	0.0001	0.0000	-0.0006	-0.0004
	HII	0.0003	0.0001	0.0000	0.0002	0.0004
	Continent [N. Am.][a]	0.0010	0.0005	0.0606	0.0000	0.0021
	T° trend : Mean temperature	0.0176	0.0018	0.0000	0.0141	0.0210
	T° trend : HII	-0.0098	0.0018	0.0000	-0.0133	-0.0063
	R^2_m	0.0119				
	R^2_c	0.0170				
Lepidoptera	Intercept	0.0207	0.0009	0.0000	0.0189	0.0225
	T° trend	-0.0541	0.0215	0.0121	-0.0962	-0.0120
	Mean temperature	-0.0003	0.0001	0.0000	-0.0004	-0.0002
	HII	-0.0002	0.0001	0.0001	-0.0003	-0.0001
	Continent [N. Am.][a]	0.0006	0.0007	0.3404	-0.0007	0.0019
	T° trend : Mean temperature	0.0103	0.0018	0.0000	0.0068	0.0138
	T° trend : HII	0.0017	0.0014	0.2293	-0.0010	0.0044
	R^2_m	0.0144				
	R^2_c	0.0288				

Table S1 (b), continued

Taxon	Variable	β	SE	P value	Lower CI95%	Upper CI95%
Lumbricidae	Intercept	0.0071	0.0003	0.0000	0.0064	0.0077
	T° trend	0.0125	0.0083	0.1344	-0.0038	0.0288
	Mean temperature	0.0001	0.0000	0.1099	0.0000	0.0002
	HII	0.0000	0.0000	0.4474	-0.0001	0.0000
	Continent [N. Am.][a]	0.0002	0.0002	0.2245	-0.0001	0.0005
	T° trend : Mean temperature	-0.0018	0.0014	0.1994	-0.0047	0.0010
	T° trend : HII	0.0004	0.0008	0.6563	-0.0013	0.0020
	R^2_m	0.0067				
	R^2_c	0.0143				
Rodentia	Intercept	0.0371	0.0063	0.0000	0.0247	0.0495
	T° trend	-0.3175	0.0762	0.0000	-0.4669	-0.1680
	Mean temperature	-0.0023	0.0003	0.0000	-0.0029	-0.0017
	HII	0.0001	0.0003	0.7034	-0.0004	0.0006
	Continent [N. Am.][a]	0.0045	0.0058	0.4460	-0.0070	0.0159
	T° trend : Mean temperature	-0.0126	0.0071	0.0783	-0.0265	0.0014
	T° trend : HII	0.0068	0.0078	0.3822	-0.0084	0.0220
	R^2_m	0.1086				
	R^2_c	0.3422				
Urodela	Intercept	-0.0009	0.0020	0.6669	-0.0048	0.0031
	T° trend	0.2161	0.0599	0.0003	0.0986	0.3336
	Mean temperature	0.0003	0.0001	0.0053	0.0001	0.0005
	HII	0.0000	0.0001	0.8840	-0.0002	0.0002
	Continent [N. Am.][a]	-0.0015	0.0011	0.1899	-0.0037	0.0007
	T° trend : Mean temperature	-0.0126	0.0036	0.0004	-0.0197	-0.0056
	T° trend : HII	-0.0014	0.0029	0.6175	-0.0071	0.0042
	R^2_m	0.0097				
	R^2_c	0.0693				

[a] Europe taken as reference

Figure S1: Spatial distribution of occurrence density in each sliding window, for each of the five time periods considered. For simplicity, results are represented as rasters with grid cells corresponding to the centroid of each sliding window.

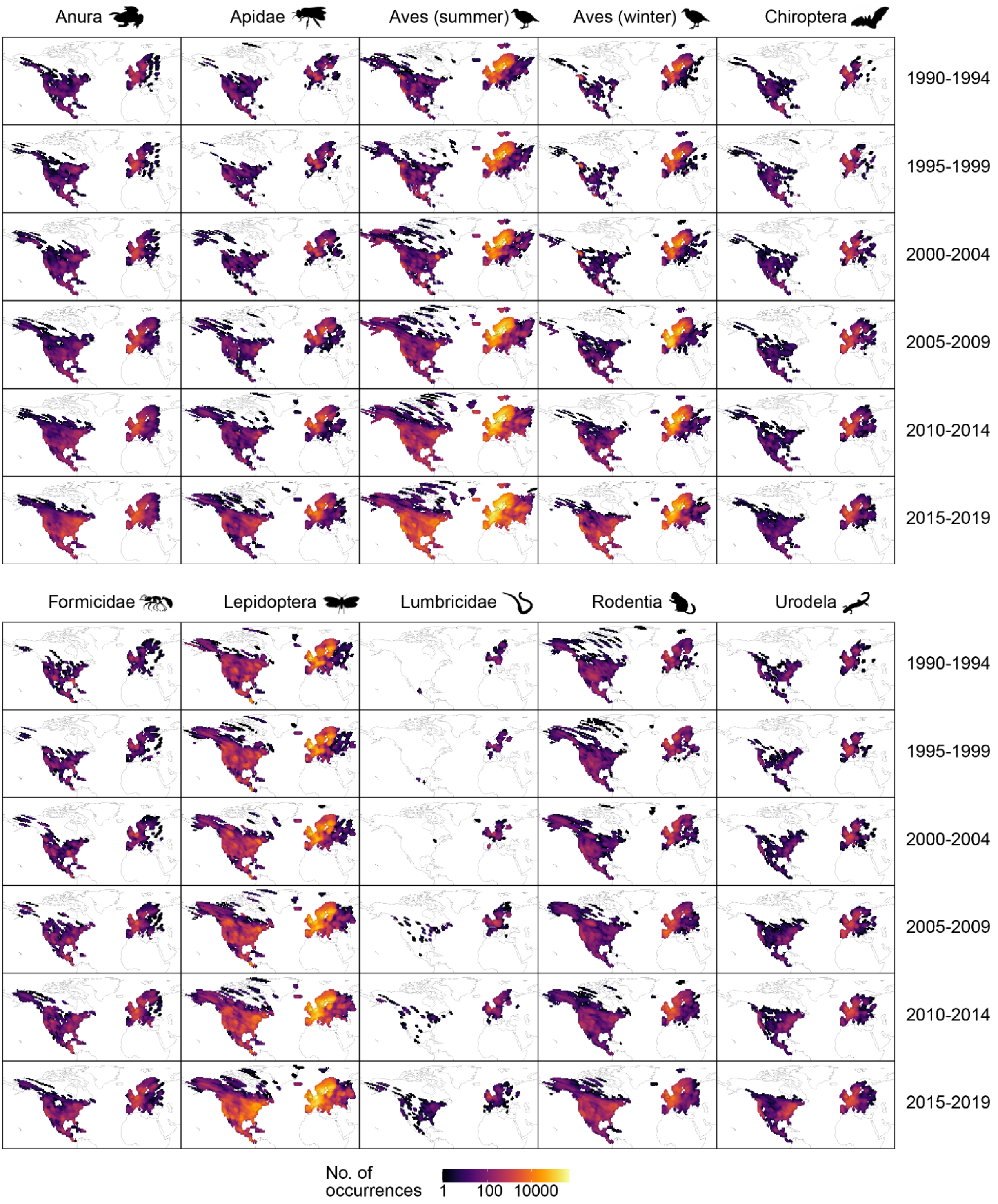

Figure S2: Spatial variation of CTI temporal trend for each taxon studied. CTI trends were extracted from the random slopes of the linear mixed model linking CTI and time (shown in Fig. 3a). For simplicity, results are represented as rasters with grid cells corresponding to the centroid of each sliding window.

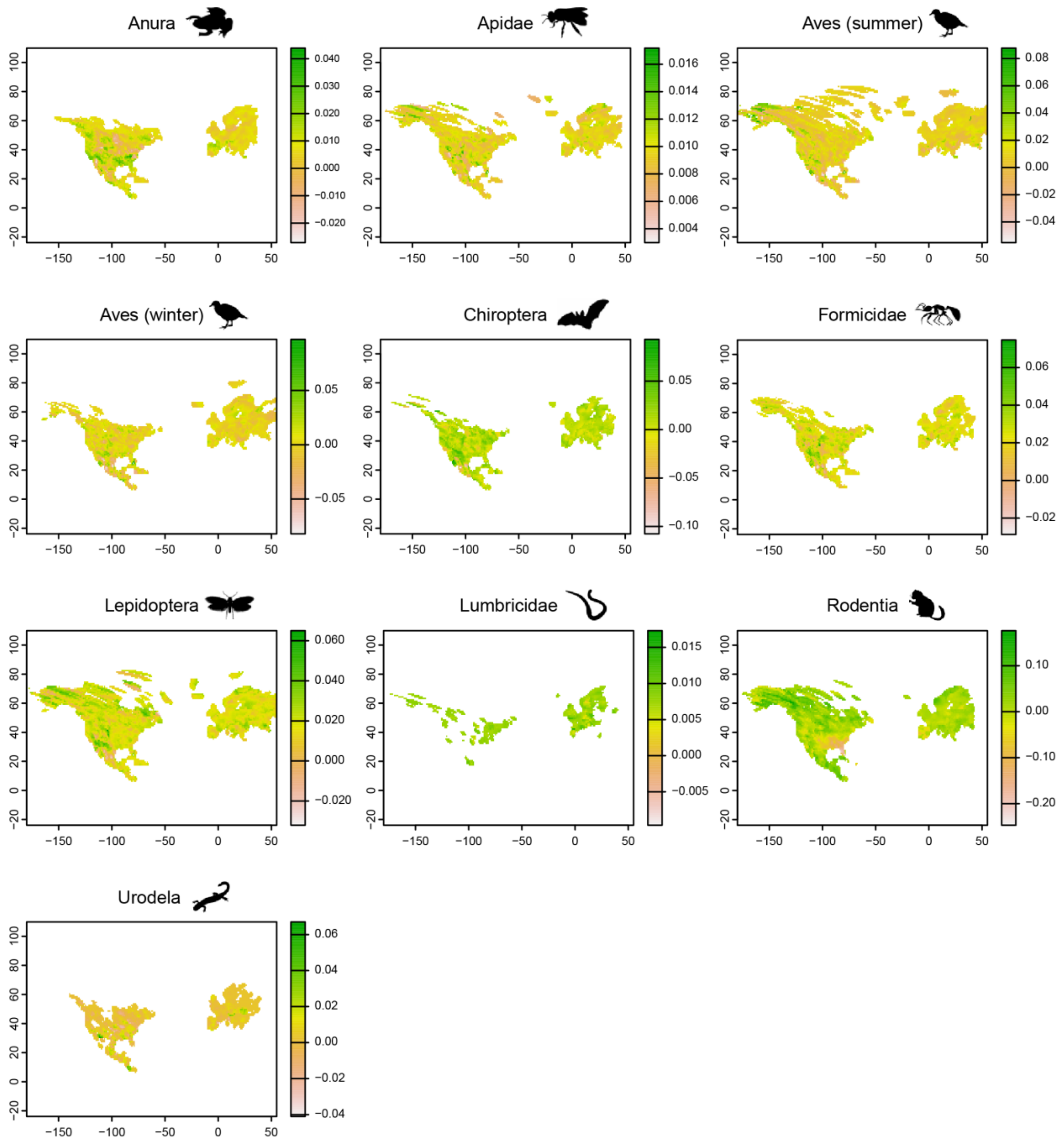

Figure S3: Distribution histograms of the differences between CTI temporal trends (at the scale of each sliding window) estimated from the filtered and from the full GBIF datasets. A negative value means that selecting only the sliding windows that contain a minimum number of years or occurrences leads to a lower estimate of the temporal trend in CTI.

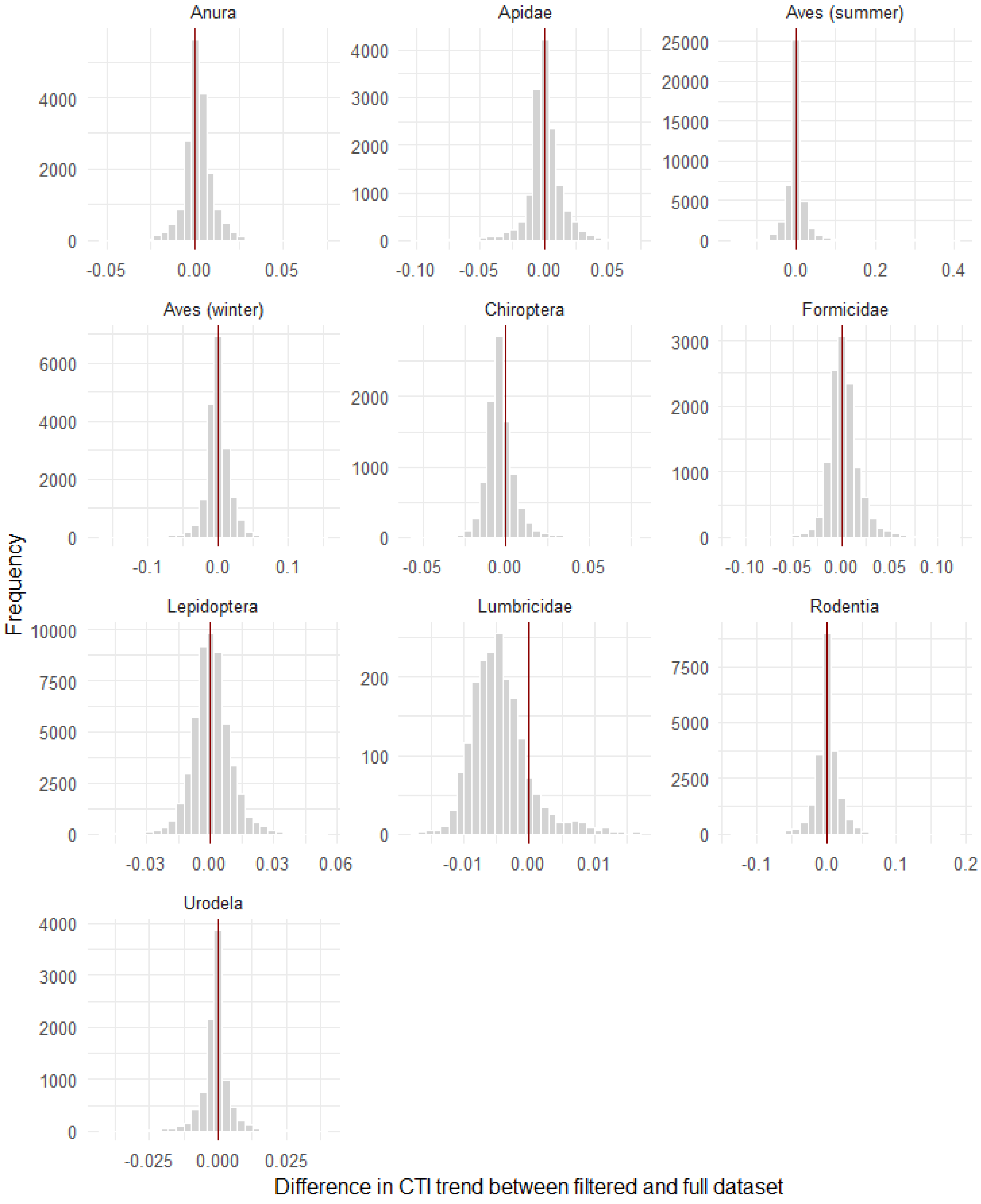

Supplement: Supplementary file 1 — Data S1. [file JANE-92-391-s001.pdf]
